# Diet and physical activity practices of South Australian adolescents

**DOI:** 10.1016/j.heliyon.2020.e04326

**Published:** 2020-08-25

**Authors:** Jason Blunt, Julia Morris, Josh Trigg

**Affiliations:** Behavioural Research and Evaluation Unit, Cancer Council SA, 202 Greenhill Rd, Eastwood, Adelaide, South Australia, 5063, Australia

**Keywords:** Nutrition, Public health, Physical activity, Health education, Applied psychology, Health psychology, Well-being

## Abstract

Adequate nutrition and physical activity are integral to health across the life course, with adolescence a crucial time for establishing health behaviours. This report describes self-reported dietary and physical activity behaviours of South Australian adolescents aged 12–17 years (*N* = 1324) surveyed in 2017. Healthy lifestyle behaviour engagement was low, with most (90.3%) adolescents not meeting Australia's recommended dietary or physical activity guidelines. Almost three-quarters (73.8%) of adolescents consumed the recommended daily amount of fruit. However, only 10.8% of adolescents met the recommended daily intake of vegetables, and large proportions regularly consumed unhealthy snacks (64.5%), fast foods, (30.7%) and sugary drinks (65.8%). Combined with the low adherence to physical activity guidelines, these findings highlight the need for effective targeted health promotion campaigns to improve adolescent's public health outcomes in South Australia.

## 1 Introduction

Maintaining a balanced diet and regular physical activity are two essential behaviours for achieving optimal health and wellbeing, making them major public health targets [1]. Research has consistently demonstrated a link between nutrition and physical activity and improved physical health in terms of a reduced chronic disease risk, decreased risk of premature death, and greater resistance to infection [1, 2, 3]. Additionally, these behaviours are associated with improved mental health, as research supports the role of diet quality and regular physical activity in mitigating common mental health disorders [4].

Adolescence is a crucial developmental period, bringing multiple physiological and psychological changes that affect nutritional needs and dietary habits [5, 6]. This is also a time when individuals’ assume greater responsibility for their own eating habits and develop health attitudes and behaviours [7]. Establishing healthy nutrition and physical activity behaviours during this time is crucial to meeting developmental needs, and to increase the likelihood of such behaviours being sustained across the lifespan.

Current guidelines from the Australian National Health and Medical Research Council (NHMRC) recommend that adolescents consume two serves of fruit and five serves of vegetables daily, perform 60 min of moderate-to-vigorous physical activity each day, and limit junk foods, snacks and sugary drink consumption [1]. Despite these guidelines and evidence highlighting the benefits of diet and physical activity across the lifespan, longitudinal data show Australians of all ages are significantly underachieving in both domains [8, 9]. Data also indicate that Australians do not eat enough fruit or vegetables, overconsume energy-dense foods containing sugar, saturated fat and sodium, and are too sedentary [8, 9]. Further, research shows that Australians are more overweight than ever before, which is largely attributed to the sustained energy imbalance caused by a lack of physical activity in combination with increased energy intake from food and drink consumption [10]. These findings and their combined health risk factors represent a burden to individuals and to Australia's health care system [11].

Understanding adolescents’ dietary and physical activity practices is important in critiquing current initiatives and shaping future policy and practice in public health. However, there is a lack of recent data on such behaviours in an Australian context. This report seeks to address this gap by providing a snapshot of healthy lifestyle practices among South Australian adolescents aged 12–17 years.

## 2 Methods

Health behaviours of South Australian adolescents were examined in 2017 via the triennial Australian Secondary Students Alcohol and Drug (ASSAD) survey. South Australian students from school Years 7–12 were recruited from public and private schools, using stratified random sampling via state-wide Australian Census school attendance records. Full methodological details (i.e. item wording) of the ASSAD survey, approved by Cancer Council Victoria's Human Research Ethics Committee, are reported elsewhere [12, 13].

### 2.1 Sample

Thirty-eight randomly-selected schools were recruited in 2017, with active parental and individual consent obtained for participating students. A total of 1,342 South Australian students aged 12–17 years were surveyed (*M* = 14.55, *SD* = 1.69), including 53.7% males and 46.3% females. Data were weighted by participant age, gender and educational sector.

### 2.2 Measures

Students reported the number of serves of fruit and vegetable they usually ate each day, the number of fast food meals they had eaten in the last week (e.g. McDonald's, KFC, take-away shops), the number of times in the past week they had consumed one serve of unhealthy snacks (e.g. a chocolate bar, a packet of chips, ice-cream, 3–4 sweet biscuits), and the number of serves of sugar-sweetened beverages (e.g., can of soft drink, sports drink, fruit juice). In this study, cut offs of ≥2 meals of junk food, ≥3 snacks, and ≥3 sugary drinks per week were applied to indicate unhealthy levels of consumption [12, 13].

### 2.3 Statistical analysis

Variations in diet and physical activity behaviours were analysed using cross-tabulation and Chi-square tests in IBM SPSS (v.23.0). Frequencies of behaviours were compared by gender and age: younger (12–15 years) versus older (16–17 years). This age split has been previously shown to reflect growing independence and increased ability to control diet and physical activity with age [13]. Responses are reported as percentages, chi-square tests, effect sizes, and significance values.

## 3 Results

### 3.1 Vegetable and fruit consumption

Table 1 shows that the majority of South Australian adolescents did not meet the recommended five daily serves of vegetables (89.5%). Although there was no significant difference between males (11.5%) and females (9.9%) in meeting the recommended daily vegetable consumption, a significantly greater proportion of older (13.1%) compared to younger (9.5%) adolescents did meet this guideline.

**Table 1:** Self-reported diet and physical activity behaviours of South Australian adolescents in 2017 (*N* = 1,342).

	*n*	%	χ^2^	*p*	ϕ
*Five serves of vegetables*					

Males	80	11.5	0.88	.349	.03
Females	61	9.9			
Younger adolescents (12–15 years)	82	9.5	**3.92**	**.048**	**.06**
Older adolescents (16–17 years)	58	13.1			
Total	141	10.8			

*Two serves of fruit*					

Males	508	73.0	**5.97**	**.015**	**-.07**
Females	483	78.8			
Younger adolescents (12–15 years)	666	77.0	2.50	.114	.04
Older adolescents (16–17 years)	325	73.0			
Total	991	73.8			

*Sixty minutes of daily physical activity*					

Males	140	20.1	**20.55**	**<.001**	**.13**
Females	67	10.9			
Younger adolescents (12–15 years)	156	18.1	**9.10**	**.003**	**.08**
Older adolescents (16–17 years)	52	11.6			
Total	208	15.9			

*Junk food*					

Males	224	32.0	1.23	.268	.03
Females	180	29.2			
Younger adolescents (12–15 years)	238	27.4	**13.09**	**<.001**	**-.10**
Older adolescents (16–17 years)	166	37.1			
Total	404	30.7			

*Sugary drinks*					

Males	266	38.2	**21.37**	**<.001**	**.13**
Females	161	26.2			
Younger adolescents (12–15 years)	266	30.8	3.78	.052	-.05
Older adolescents (16–17 years)	161	36.1			
Total	427	32.6			

*Snacks*					

Males	443	63.6	0.65	.422	-.02
Females	402	65.7			
Younger adolescents (12–15 years)	562	65.1	0.42	.516	.02
Older adolescents (16–17 years)	283	63.3			
Total	845	64.5			

*Note*. Table values bolded if test significance *p* < .05.

The majority of adolescents consumed the recommended two or more serves of fruit per day (73.8%). A small yet statistically greater proportion of males met this guideline (73.0%) compared to females (78.8%). There was no statistical difference between younger (77.0%) and older (73.0%) adolescent fruit consumption (Table 1).

An optimal diet was assessed as meeting both the fruit and vegetable guidelines over the previous week, with 9.7% (*n* = 131) of students achieved an optimal dietary outcome. Of participants with optimal diet, more were male (57.7%, *n* = 75) than female (42.3%, *n* = 55), although this difference was not statistically significant (χ^2^ (1, 1342) = 0.91, *p* = .340, ϕ = -.02). There was no significant difference between younger adolescents (58%, *n* = 76) and older adolescents (42%, *n* = 55) (χ^2^ (1, 1342) = 3.78, *p* = .052, ϕ = -.05).

### 3.2 Physical activity

Of the 15.9% of adolescents who met the physical activity criteria over seven days, a significantly greater proportion were male (20.1%, *n* = 140) than female (10.9%). A significantly greater proportion of younger adolescents (18.1%) met this guideline compared to older adolescents (11.6%).

### 3.3 Meeting all guidelines

Twenty five adolescents (1.9%) met each of the fruit, vegetable, and physical activity guidelines for the previous week. The majority were male (68.0 %, *n* = 17) and aged 12–15 years (72.0%, *n* = 18). However, differences by gender (χ^2^ (1, 1342) = 2.10, *p* = .147, ϕ = -.04) and age (χ^2^ (1, 1342) = 0 .45, *p* = .501, ϕ = -.02) were not significant.

### 3.4 Discretionary food consumption

#### 3.4.1 Junk food

The NHMRC recommended limit of two serves of junk food per week for adolescents [1] was met or exceeded by 30.7% of the sample. No significant gender differences were found in junk food consumption. However, a significantly greater proportion of older adolescents (37.1%) consumed two or more junk food meals per week compared to their younger counterparts (27.4%) (Table 1).

#### 3.4.2 Snacks

Adolescents are advised to limit snack consumption to less than three serves per week. However, 64.5% of adolescents met or exceeded this limit in 2017. There were no significant differences between ages or gender in snack consumption (Table 1).

#### 3.4.3 Sugary drinks

In 2017, 65.8% of adolescents consumed sugary drinks three or more times per week. Although there were no significant age differences in consumption, a significantly greater proportion of males (38.2%) consumed three or more serves of sugary drinks per week compared to females (26.2%).

## 4 Discussion

This report described South Australian adolescents’ adherence to Australian dietary and physical activity guidelines. Results indicate that adolescents are not meeting their recommended intake of vegetables, are too inactive, and consume too many energy-dense discretionary foods and drinks.

Older adolescents had significantly lower levels of physical activity and a higher self-reported junk food intake than younger adolescents. These findings are consistent with previous literature reporting that health-promoting exercise tends to decline as individuals enter adolescence [14]. Research suggests that this decline reflects external pressures such as academic load, part-time jobs, and peer group maintenance, which often hinder health behaviour engagement during adolescence [13, 15]. The current findings indicate that further policy intervention is needed to support physical activity and healthy diet in South Australian adolescents, particularly during the latter stages of this formative period. Resources that promote the adoption of health behaviours during adolescence have been found to promote healthier behaviour trajectories into adulthood [16]. Thus, interventions designed to motivate and assist adolescents adopt healthy behaviours during this transitional period will likely have long-term health benefits, such as the prevention of chronic disease and mental health disorders [1, 2, 3, 4, 17].

In combination with poor dietary practices, the majority of adolescents surveyed did not meet the recommended physical activity guideline of at least 60 min physical activity every day [1]. Consistent with current literature, physical activity levels were significantly lower in female and older adolescents [15]. Increasing female engagement in physical activity is complex, with low participation arising from a number of factors such as normative pressures, body image, and limited gender equality promotion in sports and physical activity spaces [18]. Despite these factors, the continued findings of low rates of physical activity in adolescent females in South Australia reinforces the need for interventions to educate, reduce barriers, and invest in supporting gender equality in physical activity programs to encourage the healthy adoption and maintenance of physical activity practices throughout the lifespan [13].

Although data were obtained from a representative sample of South Australian adolescents, it is unclear the extent to which these findings can be applied to adolescents nationally. No data were available regarding adolescents’ attitudes towards diet and physical activity, and it could not therefore be determined whether recommended intake guidelines were known or understood. Further research into this additional potential barrier is warranted to support adolescents through targeted policy and interventions to achieve optimum diet and physical activity.

This report on the dietary and physical activity behaviours of a representative sample of South Australian adolescents adds weight to the argument that habits and behaviours formed in adolescence can predispose younger Australians to a lifestyle of poor diet and exercise, thereby increasing health and disease risk in adulthood. If, as evidence suggests, diet and physical activity habits established in the formative years reassert themselves throughout the lifespan, adolescence marks a definitive period for intervention and policy to establish healthy psychological and physical systems of behaviour to prevent poor lifestyle behaviours form developing, becoming ingrained and, therefore, harder to modify as a person ages.

## Declarations

### Author contribution statement

J. Blunt: Conceived and designed the experiments; Performed the experiments; Analyzed and interpreted the data; Contributed reagents, materials, analysis tools or data; Wrote the paper.

J. Trigg and J. Morris: Conceived and designed the experiments; Analyzed and interpreted the data; Wrote the paper.

### Funding statement

This work was supported by the Australian Department of Health and Cancer Council SA.

### Competing interest statement

The authors declare no conflict of interest.

### Additional information

No additional information is available for this paper.

## Appendix A Supplementary data

HELIYON-D-19-02728 supplementary material HELIYON-D-19-02728 supplementary material
