# Supplementary material for: Diet and physical activity practices of South Australian adolescents
Source: Heliyon. 2020 Aug 25;6(8):e04326. doi: 10.1016/j.heliyon.2020.e04326 (PMC7452447; doi:10.1016/j.heliyon.2020.e04326)
Supplement: HELIYON-D-19-02728 supplementary material [file mmc1.docx]

The following questions were administered as a part of a larger survey of South Australian secondary school students, assessing attitudes and behaviours towards alcohol and drug use, sun, nutrition, and social media behaviours. The questions listed were utilised to obtain the data described in the paper, “Diet and physical activity practices of South Australian adolescents”.

**THE NEXT FEW QUESTIONS ARE ABOUT FOOD YOU MIGHT HAVE EATEN.**

75. How many **serves** of vegetables do you usually eat **each day?** *(A serve is equal to ½ cup of cooked vegetables or 1 cup of salad vegetables, or ½ a cup of cooked dried beans, lentils, chickpeas, split peas or canned beans)*

| 1ρ1 serve or less | 3ρ3 serves | 5ρ5 serves | 7ρI do not eat vegetables |
| --- | --- | --- | --- |
| 2ρ2 serves | 4ρ4 serves | 6ρ6 serves or more |  |

76. How many **serves** of fruit do you usually eat **each** **day?** *(A serve is equal to
1 medium piece or 2 small pieces of fruit, or 1 cup of diced pieces of fruit, or 4 dried apricot halves, or ½ a cup of juice)*

| 1ρ1 serve or less | 3ρ3 serves | 5ρ5 serves | 7 I do not eat fruit |
| --- | --- | --- | --- |
| 2ρ2 serves | 4ρ4 serves | 6ρ6 serves or more |  |

**THE NEXT FEW QUESTIONS ASK ABOUT WHAT YOU DID IN THE LAST WEEK.**

77. How many times **in the last week** have you eaten meals that were bought from fast food outlets like *McDonalds, Hungry Jacks, Pizza Hut, KFC, Red Rooster, hamburger or fish and chip shops*?

| 1 None | 3 2 times | 5 4 times |
| --- | --- | --- |
| 2 Once | 4 3 times | 6 5 or more times |

78. How many times **in the last week** did you eat **snacks** like *a chocolate bar, a piece of cake, a packet of chips/twisties/corn chips, icecream, 3–4 sweet biscuits?*

| 1 None | 3 2 times | 5 4 times |
| --- | --- | --- |
| 2 Once | 4 3 times | 6 5 or more times |

79. How many times **in the last week** did you drink a can of **soft drink** (*like Coke, Pepsi, lemonade, Fanta), a sports drink (like Powerade, Gatorade), an* ***energy drink*** *(like Redbull, V, Wild),* ***fruit juice*** *or* ***have at least 2 glasses of cordial in a row****?* This does not include diet or low joule drinks.

| 1 None | 3 2 times | 5 4 times |
| --- | --- | --- |
| 2 Once | 4 3 times | 6 5 or more times |

**THE NEXT FEW QUESTIONS ARE ABOUT PHYSICAL ACTIVITY.**

80. How many days **in the past week** have you done any **moderate** or **vigorous** physical activity for a **total of at least 60 minutes**? (This doesn't have to be all in one go it could be made up of different activities during the day like cycling or walking to and from school, playing sport at lunchtime or after school, doing an exercise class, doing housework etc)

| 1 1 day | 4 4 days | 7 7 days |
| --- | --- | --- |
| 2 2 days | 5 5 days | 8 No days in the last week |
| 3 3 days | 6 6 days |  |
